# Comprehensive assessment for different ranges of battery electric vehicles: Is it necessary to develop an ultra-long range battery electric vehicle?

**DOI:** 10.1016/j.isci.2023.106654

**Published:** 2023-04-20

**Authors:** Xinglong Liu, Fuquan Zhao, Jingxuan Geng, Han Hao, Zongwei Liu

**Affiliations:** 1 State Key Laboratory of Automotive Safety and Energy, Tsinghua University, Beijing 100084, China; 2 Tsinghua Automotive Strategy Research Institute, Tsinghua University, Beijing 100084, China; 3 China Automotive Energy Research Center, Tsinghua University, Beijing 100084, China

**Keywords:** Electrochemical energy storage, Energy resources, Energy management, Energy modeling

## Abstract

Some automotive companies develop battery electric vehicles (BEVs) with an ultra-long range to address consumers’ range anxiety. However, ultra-long-range BEVs have many problems, and whether they can truly solve consumers’ range anxiety has not been studied. Thus, we build a technology-rich, bottom-up approach model to evaluate BEVs’ performance, economy, and total cost of ownership (TCO) to reveal the necessity of developing ultra-long-range BEVs. The results show that the ultra-long-range BEVs’ dynamic, safety, and economy performances are poor compared to short-range BEVs. Based on the TCO analysis considering battery replacement and alternative transportation costs, 400 km is the optimal range of BEVs for consumers. In addition, consumers’ range anxiety is essentially anxiety about energy replenishment. Ultra-long-range BEV cannot really solve consumers’ range anxiety except by reducing charging frequency. In the case of gradually improving the charging and swapping infrastructure, we believe that automotive companies do not need to develop ultra-long-range BEVs.

## Highlights

• The vehicle characteristics of BEV with different ranges are calculated by TEMP
• TCO model considering the battery degradation and alternative transportation is built
• The necessity of developing and promoting the ultra-long-range BEV is studied
• Some policy suggestions for solving the consumer’s range anxiety are proposed

## Graphical abstract

**Figure:**
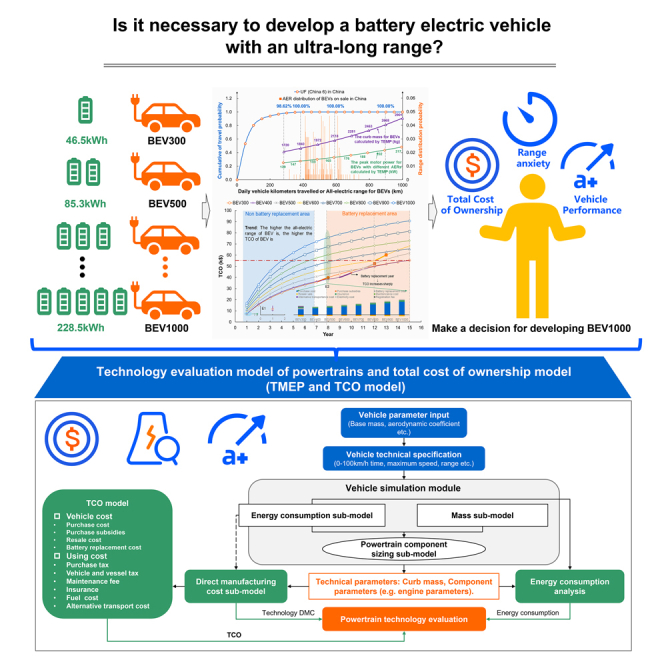

## Teaser

Electrochemical energy storage; Energy resources; Energy management; Energy Modelling

## Introduction

China’s carbon neutrality goal has accelerated the clean transformation of the automotive industry to reduce greenhouse gas emissions.^1^^,^^2^^,^^3^ A series of policies and regulations formulated by the Chinese government put higher requirements on automotive companies to achieve energy saving and emission reduction.^4^ Developing new energy vehicles (NEVs) has become the main path to meeting the regulatory requirements and achieving energy conservation and emission reduction for automotive companies.^5^^,^^6^ According to the China Association of Automobile Manufacturers, the number of NEVs in China was about 13.10 million, of which battery electric vehicles (BEVs) accounted for about 79.78% by December 2022.^7^ The development of BEV has changed from policy-oriented to market-oriented. However, it does not mean that the development of BEVs has been unimpeded. According to a survey conducted by PricewaterhouseCoopers among 3,000 Chinese, American, and German consumers, range anxiety and inadequate charging infrastructure are the main reasons consumers abandon the purchase of BEVs. In China, short all-electric range (AER) and inadequate charging network coverage are the biggest obstacles to the popularity of BEVs.^1^

To enhance BEVs’ competitiveness and accelerate their products’ promotion, automotive companies adopt different approaches to solve the consumers’ range anxiety. Firstly, some automotive companies accelerate the research and development (R&D) of fast-charging technologies and the layout of fast-charging stations. They hope to quickly replenish BEVs through fast-charging technologies like Tesla and Xpeng.^8^ Secondly, some automotive companies have developed battery-swapping technology to solve the problem of fast energy replenishment for BEVs, realizing a quick battery swap within 3–5 min. One of the better automotive companies is NIO.^9^ Finally, some automotive companies use the way to improve the AER (by adding the battery capacity) to solve consumers’ range anxiety. For example, GAC Aion and NIO have announced to develop the BEVs with 1000 km AER (BEV1000).^10^ Due to the lack of a breakthrough in fast-charging technology and insufficient charging infrastructure, developing BEVs with ultra-long AER has become the mainstream way automotive companies solve consumers’ range anxiety.

However, what is the nature of consumers’ range anxiety, and can the BEV with an ultra-long range truly solve it? It is a question that is well worth studying. Compared to BEVs with short AER, the BEV with an ultra-long range has advantages and disadvantages. For example, the most significant advantage of the BEV1000 is its long AER, which can satisfy consumers’ long-distance travel needs without worrying about the range anxiety and the charging anxiety of finding charging piles. It is especially important during holidays when consumers travel long distances, and they all have the charging requirement. The second advantage of BEV1000 is the relatively large battery capacity, which can meet the travel needs of urban consumers for almost a month on a single charge. It could reduce the hassle of frequent charging for consumers. Unfortunately, a key challenge is whether the BEV with an ultra-long range can truly address consumers’ range anxiety at the cost of a higher incremental cost (with a larger battery) and reduced performance (dynamic, braking performance, and higher energy consumption) compared to a BEV with a shorter AER?

**Figure 1:**
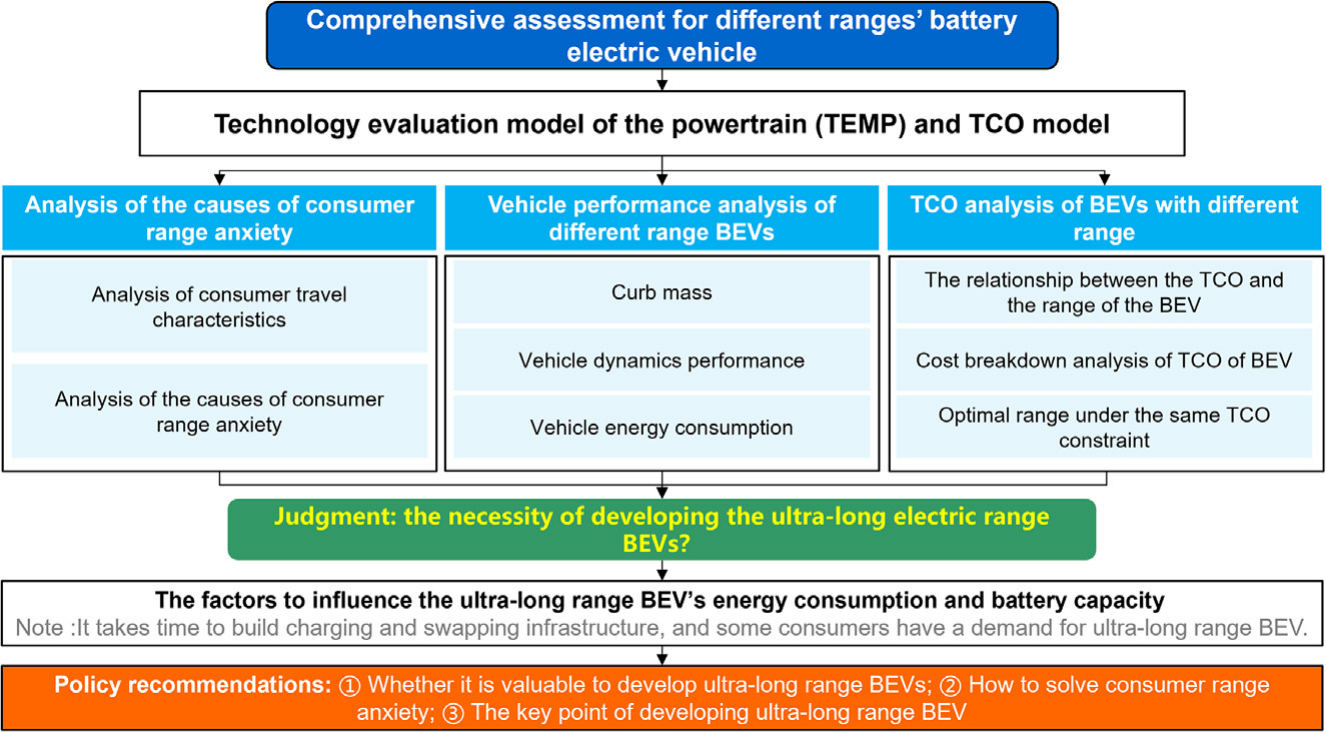
The research framework of this paper

Previous literature has extensively studied the impact of increasing AER on the economics and environment of BEVs and also explored the relationship between AER and consumers’ range anxiety. For example, Zhang et al. compared BEVs’ life cycle greenhouse gas emissions with different AERs.^11^ They found that BEVs in 2020 with an electric range over 700 km are even worse in carbon emission, the worst is −33.08% of BEV1000. Zachary et al. investigated the potential for widespread electrification of personal vehicle travel in the United States, considering the range anxiety of BEV.^12^ They found that an existing, affordable electric vehicle could meet the energy requirement of 87% of vehicle day. They suggested that other vehicle technologies are likely to be needed even as batteries improve and charging infrastructure expands to solve the range anxiety of BEV. Yang studied the effect of AER on the BEV coupling cost and energy efficiency.^13^ They found that with the increase of AER, the total cost increases while the energy efficiency decreases regardless of battery type. However, there is relatively little research on the comprehensive evaluation of the BEV with an ultra-long range. Here, this paper analyzes the BEV with an ultra-long range from four aspects: the essence of consumers’ range anxiety, vehicle performance, the total cost of ownership (TCO) for consumers, and the proper development mode. This paper explores the necessity of developing the BEV with an ultra-long range and the technology direction of improvement. The research framework is as shown in Figure 1.

The main innovation of this study is to comprehensively evaluate the necessity of developing ultra-long-range BEVs from the three perspectives of range anxiety analysis, vehicle performance analysis, and TCO analysis. It can solve the policy concerns of automakers, consumers, and the government. However, few current studies systematically answer this question from these three perspectives. Meanwhile, this study also conducts a brief parameterization study on the TCO to suggest pathways for improving BEVs’ TCO. A technology-rich, bottom-up approach model is established to calculate the component parameters of BEVs with different AERs and to evaluate the vehicle performance, manufacturing cost, and TCO. The results show that the essence of consumers’ range anxiety is the energy replenishment anxiety of BEVs. BEV with an ultra-long range only reduces the charging frequency, which does not improve the energy replenishment speed and does not really solve consumers’ range anxiety. With the charging infrastructure gradually improving, companies do not need to develop BEV with an ultra-long range. Suppose there is a need to develop BEV with an ultra-long range to meet the needs of some special consumers. In that case, more efforts should be made to improve the energy density of batteries and use advanced vehicle energy-saving technologies.

## Results and discussion

### Ultra-long-range BEV does not really solve consumers’ range anxiety

**Figure 2:**
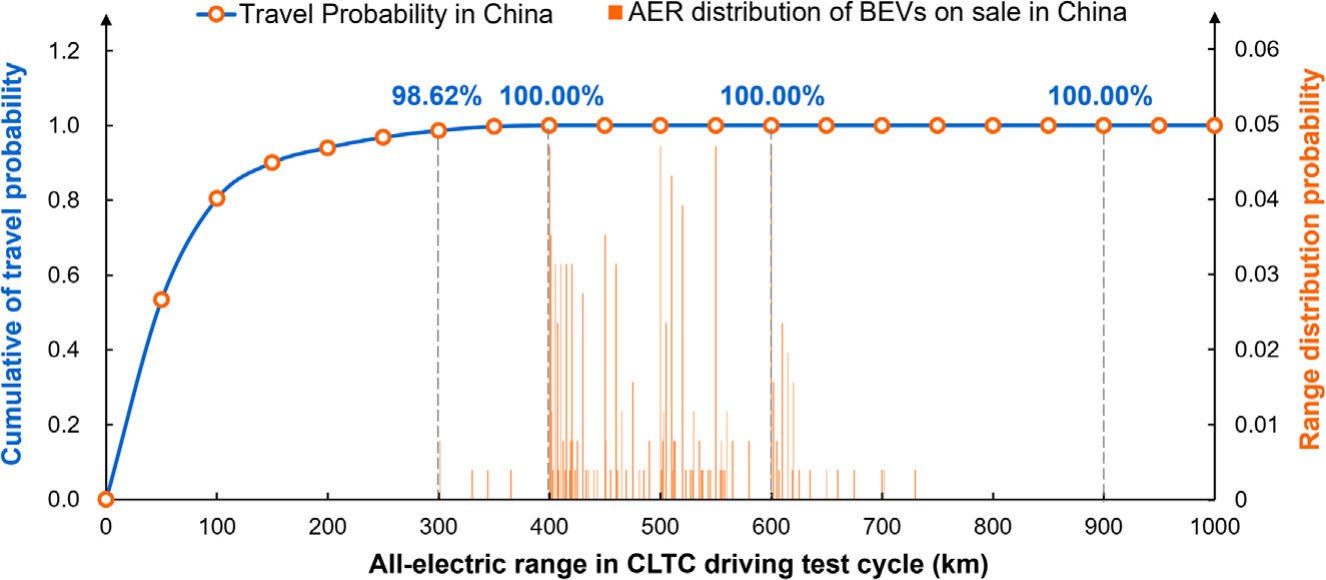
The cumulative probability distribution of daily travel range for consumers and the AER distribution of BEVs (B segment) on sale in China

As shown in Figure 2, the blue line depicts the cumulative probability distribution of daily travel mileage for consumers in China.^14^ It can be seen that travel mileage below 300 km occupies 98.62% of people’s travel scenarios. It means that BEV300 can satisfy 98.62% of people’s daily travel scenarios. In addition, it can be seen that when the daily mileage of 400 km could basically cover all the daily travel scenarios of people in China. Therefore, when the BEVs’ AER exceeds 400 km, there is no range anxiety in single-day travel from the perspective of the probability of people’s daily travel mileage. Of course, this is only statistical data and cannot cover all travel scenarios. Based on the above-mentioned description, if there is a slow-charging pile at home, it is sufficient to replenish the battery every time, which is also good for the battery for 400 km BEV. The orange squares represent the current AER distribution of B-segment BEVs on sale in China. The AER of B-segment BEVs sold in China is mainly in the 400–600 km range, especially in the 400–500 km range. It can be said that the current mainstream BEVs can match more than 98% of consumers’ travel needs. In the case of gradually improving charging infrastructure, BEVs can achieve a daily charge and match travel needs. Then, it is not very meaningful for automotive companies to develop BEVs with a range of 1000 km and above.

However, why does range anxiety exist for BEVs’ consumers? We guess consumers’ range anxiety mainly comes from two aspects: First, one cause of range anxiety could be that the real range of BEVs differs greatly from the nominal AER announced by automotive companies. Especially when the air conditioning is on, and the battery performance decreases in winter, it results in a lower real range. For example, the real range of BEVs is approximately 35%–40% lower than the nominal range (at minus 7°) in cold regions of China.^15^ BEV1000 can still partially address this anxiety due to its higher AER, even if an inflated true AER exists. However, if less power is left in the BEV1000, consumers will still have some range anxiety. The second reason we guessed for consumers’ range anxiety is charging anxiety. It is mainly due to the imperfect charging infrastructure, consumers’ inability to find charging piles in time, or charging time being too long, especially when battery charging is not on the consumers’ schedule. Data from the China Electric Vehicle Charging Infrastructure Promotion Alliance show that, of the approximately 3,651,000 vehicles sampled with pile-by-pile information, the proportion of charging piles not attached to vehicles was about 13% as of November 2022.^16^ It also means that more than 13% of vehicle owners in China still need to replenish their energy at public charging sites. In addition, China’s current public charging stations are mainly 60 kW fast-charging piles, and the power of fast-charging piles in some areas is only 30 kW. It would take 3.8 h to fully charge a BEV1000. It is not a short waiting time for consumers who need to replenish energy quickly. Although ultra-long-range BEVs can reduce the charging frequency, their charging time is still similar to short-range BEVs. Both ultra-long-range and short-range BEVs will need to rely on more public charging posts and faster public charging speeds to solve that problem.

Therefore, charging anxiety is the culprit of the consumer’s range anxiety. It is well known that BEVs can be limited by their range, yet gasoline vehicles are not unlimited. Why is it that gasoline vehicles have a range of around 500 km but no consumers’ range anxiety? On the one hand, they do not have the problem of inaccurate battery SOC estimation like BEVs. Their driving ranges could not decline extremely fast in extremely cold winters. On the other hand, there are gas stations all over the place, so they can replenish fuel nearby, and the refueling time is only a few minutes. It can be said that if there is a wide, convenient, and reliable charging network, even the range of only 400 or 500 km of BEVs is sufficient to meet the travel needs of consumers. On the contrary, consumers will still have range anxiety even if the AER of BEV is 1000 km. Therefore, BEV1000 does not essentially solve consumers’ range anxiety.

### Ultra-long-range BEV has no other performance advantages than longer AER

**Figure 3:**
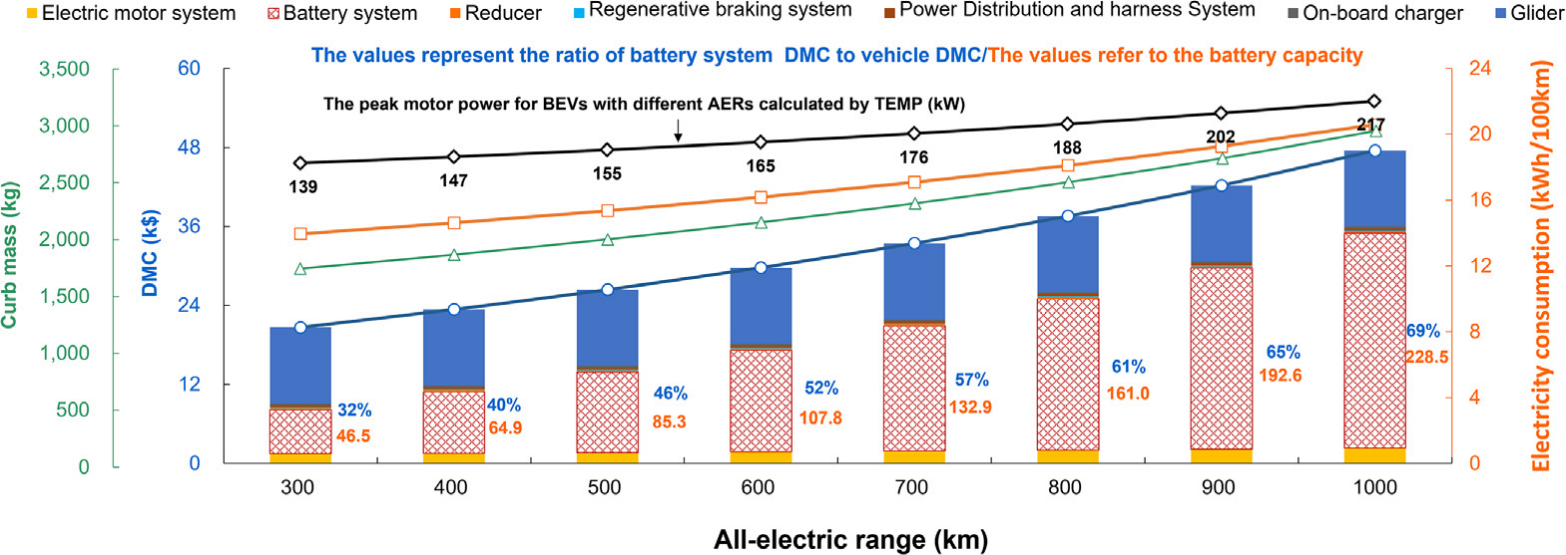
BEVs’ characteristics with different AERs including curb mass, DMC, electricity consumption, and dynamic performance

Before the revolutionary breakthrough in power battery technology, the increase of BEVs’ AER mainly relies on improving battery capacity. In this paper, the direct manufacturing cost (DMC) and component parameters of BEVs with different ranges are evaluated quantitatively with the help of the established TEMP, as shown in Figure 3.

It can be seen that with the increase of AER, the curb mass of BEV gradually increases. BEV1000’s curb mass is 68.66% higher than BEV300’s. Increase in curb mass is expected to lower the performances of BEVs in terms of power, maneuverability, braking, and economy. For example, according to TEMP calculations, the drive motor peak power of the BEV1000 (217 kW) is 56.12% higher than the drive motor peak power of the BEV300 (139 kW) to achieve the same acceleration performance. Moreover, the electricity consumption of BEV1000 (20.57 kWh/100 km) is 47.6% higher than BEV300 (13.94 kWh/100 km). It greatly reduces the energy economy of BEVs, which is not in line with the development concept of energy saving and efficiency of automotive companies.

In addition, the ultra-long-range BEVs means carrying a larger capacity battery. The battery capacity of BEV1000 is 228.5 kWh. It should be noted that the 228.5 kWh battery pack for BEV1000 can be installed by being integrated into the vehicle’s chassis (about 657L), but it may squeeze the space in the cockpit. The battery capacity of BEV1000 is about 5 and 3.5 times of the BEV300 and BEV400. In other words, the battery capacity of producing one BEV1000 can produce about 5 and 3.5 BEV300s and BEV400s. It will bring a series of social problems. With an ultra-long range, BEV will consume more battery raw materials to meet consumer travel demand. It will increase the dependence of BEVs on lithium resources, exacerbate the current lithium resource supply tension, and cause a certain degree of resource waste.^17^ Secondly, the DMC of BEV1000 will increase significantly, especially the cost of the battery system. It can be seen in the Figure 3, the battery cost of BEV300 accounts for about 32% of the vehicle cost, while the proportion of BEV1000 is as high as 69%. It will likely reduce consumers’ enthusiasm to buy BEVs, which would become an obstacle to the large-scale promotion of BEVs in China.

Thirdly, ultra-long-range BEVs are equipped with larger capacity batteries, which increase the difficulty of energy management and safety protection of power batteries.^18^ At the same time, larger power fast-charging devices are generally chosen to shorten the charging time of long-range BEVs. It will further increase the risk of thermal runaway of the vehicle battery and lead to safety situations such as spontaneous vehicle combustion.^19^ In general, BEV with an ultra-long range has no advantage other than a longer range in terms of the vehicle product itself. Moreover, it also requires more advanced design investment to achieve performance similar to shorter range BEVs.

### Ultra-long-range BEV has a high TCO and is not consumer friendly

One of the reasons why consumers prefer BEVs is their low TCO. In this paper, we calculate the TCO of BEVs, covering vehicle cost (purchase cost, purchase subsidy, vehicle residual value, and battery replacement cost) and use the cost (purchase tax, vehicle and vessel tax, registration tax, insurance, repair and maintenance cost, electricity cost, and alternative transportation cost). Among them, battery replacement cost refers to BEVs with short ranges needing to be charged more frequently to meet the annual travel demand. It leads to the degradation of battery performance and cannot meet the performance requirements of the vehicle, and then need to replace the battery with the same capacity. The alternative transportation cost refers to choosing other transportation alternatives (this paper uses ICEVs) because the BEVs’ AER cannot meet the consumer’s travel after a single charge.

**Figure 4:**
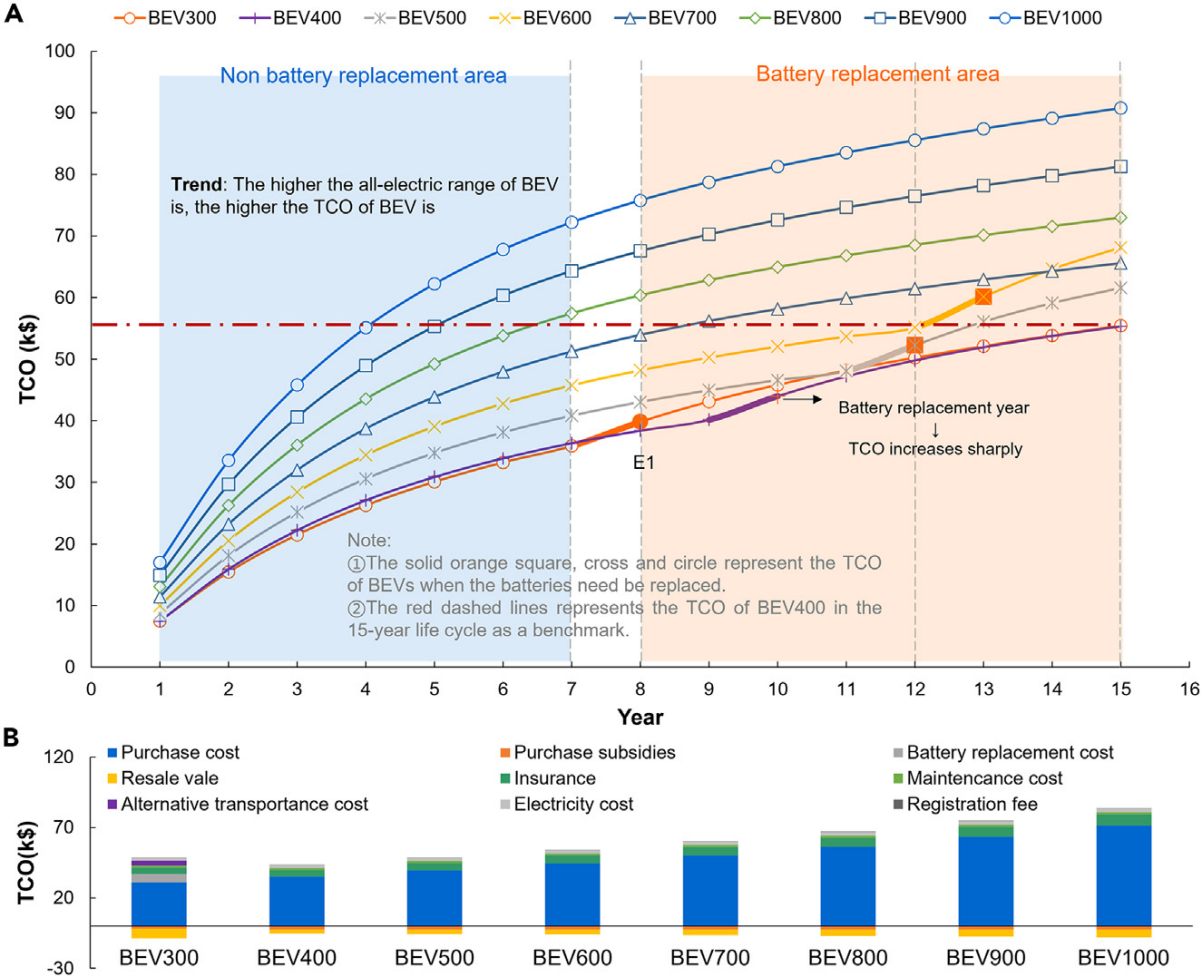
TCO analysis (A) TCO of BEVs with the different ranges at different life cycle spans. (B) TCO breakdown for the BEV400 at the point E1.

Based on the established TCO model, the TCO of BEVs with different ranges is calculated at different life cycle spans, as shown in Figure 4A. Based on the battery degradation model and the annual vehicle kilometer traveled by the vehicle, no battery replacement is required for the first 7 years for each range of BEVs. While the BEV300, BEV400, BEV500, and BEV600 need to be replaced once in the 8th, 10th, 12th, and 13th years, respectively. In the life cycle span without battery replacement, the TCO of BEV increases gradually with the increase of AER. The longer the life cycle span, the greater the increase in cost. For example, the BEV1000’s TCO is $28,018 higher in year 4 relative to the BEV400, and $35,835 higher in year 7 relative to the BEV400. There is a certain jump in TCO for BEV models with battery replacement over the life cycle span that with battery replacement, but overall, it still shows that the higher the AER, the higher the TCO of the vehicle.

The histogram in Figure 4B shows the breakdown of the TCO for BEVs with different AERs in year 8. The purchase cost of the vehicle and the insurance (related to the purchase cost) are the main sources of the increase in the TCO. Surprisingly, the electricity cost does not vary greatly across BEVs, even though the higher AER BEVs have relatively higher electricity consumption. It is mainly due to the relatively low electricity costs, and the relatively low annual vehicle kilometer traveled, which does not lead to a large difference in electricity costs. BEV300 does not experience a significant increase in its TCO after battery replacement due to the high residual value of the replaced battery in year 8. (In this paper, it is assumed that the resale value rate of the battery is equal to that of the vehicle as above-mentioned, and the residual battery value is calculated according to the VDR after one year). In general, the purchase cost is the most significant factor in the increase in the TCO of a BEV. Therefore, reducing the AER can significantly reduce the TCO of BEVs.

In addition, we use the TCO of BEV400 in the 15-year life cycle as a benchmark to analyze the service life of BEVs with different AERs, as shown by the red dotted line in Figure 4B. BEV1000 can only be used for about four years under the same TCO, and BEV900 can be used for about five years. Similar to it is that BEV300 and BEV500 can be used for about 15 years and 13 years, respectively. However, the BEV300 will reduce the convenience of travel for consumers compared to the BEV400 due to the need for a higher frequency of alternative transportation. Therefore, the optimal range for BEVs is 400 km when considering consumer range anxiety (alternative transportation cost) from the perspective of TCO. At the same time, this can further show that the AER of BEVs is not as high as possible, but choose the appropriate AER to exploit its cost-friendliness further.

### Ultra-long-range BEV’s reasonable research and development mode

**Figure 5:**
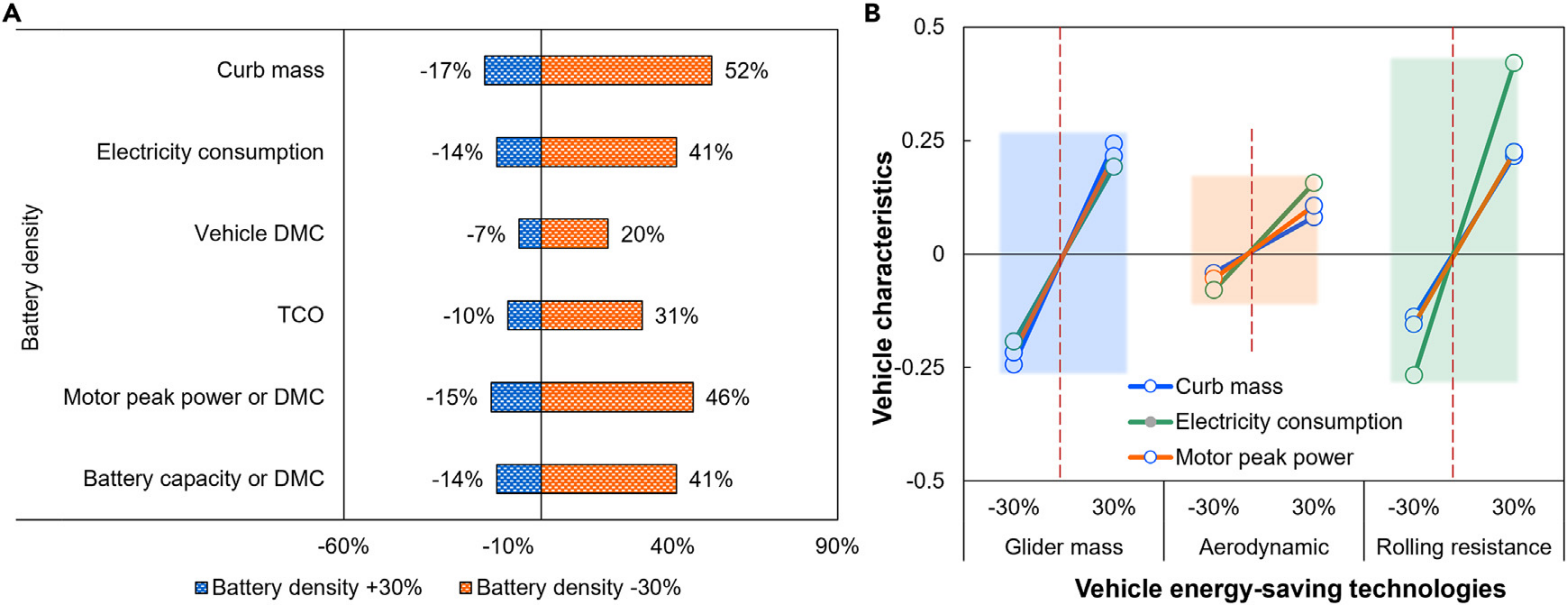
Uncertainty analysis of battery and lightweight technologies for BEV1000’s characteristics (A) Battery energy density. (B) Vehicle energy-saving technologies.

Currently, the construction of the charging and swapping infrastructure network takes a certain amount of time, and some consumers demand ultra-long-range travel. BEV1000 does have a certain market demand for some special consumers. Although BEV1000 has some problems in dynamic, braking, safety, and economic performance, some automotive companies will still develop BEV1000 to meet the needs of these consumers. How to reasonably develop 1000 km BEVs? This paper gives some quantitative analysis calculated by TEMP from two aspects of battery and vehicle energy-saving technologies and the development suggestion for BEV1000, as shown in Figure 5.

Figure 5A shows the uncertainty analysis of the impact of changes in battery energy density on the performance of the BEV1000. When the battery energy density is increased by 30%, the BEV1000’s battery capacity is reduced by 14%, and the required motor’s peak power is reduced by 15%. In turn, it leads to a 17% and 14% reduction in its curb mass and electricity consumption, respectively. When the battery energy density is reduced by 30%, the BEV1000’s battery capacity, motor’s peak power, curb mass, and electricity consumption is increased by 41%, 46%, 52%, and 41%, respectively. The above data show that improving battery energy density can reduce energy consumption and vehicle manufacturing cost. It makes its TCO lower and enhances the BEV’s acceptance of consumers. Therefore, automotive companies should make great efforts to improve battery energy density when developing BEV1000, such as researching new battery materials, developing solid-state batteries, metal-air batteries, and so on.^20^

In addition, energy-saving technologies can be adopted when developing ultra-long-range BEVs. With intelligent connected vehicle technology development, BEVs need to add more sensors, central calculators, and other components.^21^ It will make their glider mass increase further. Figure 5B shows the effect on BEV1000’s performance when the glider mass changes. It is found that when the glider mass of BEV1000 is reduced by 30%, it can make the electricity consumption, motor’s peak power, and curb mass reduced by 19%, 22%, and 24%, respectively. Conversely, the corresponding vehicle performance results are improved. Considering that the cost of vehicle light-weighting technology is challenging to obtain, this paper does not calculate the vehicle’s DMC and TCO of BEV1000. However, the above data still indicate that when automotive companies have to develop BEV1000, selecting appropriate lightweight technologies (such as body material substitution, manufacturing process optimization, etc.) is crucial for the energy saving of the vehicle.^22^ At the same time, reducing the wind resistance coefficient and rolling coefficient can also reduce the electricity consumption and battery capacity of BEV1000 as shown in Figure 5B. As shown by the boxes of different colors in Figure 5B, the technology of reducing rolling resistance to improve vehicle performance has the greatest energy-saving potential (according to the slope of the rectangular box) among these technologies. Therefore, automotive companies can choose appropriate energy-saving tires when developing BEVs with an ultra-long range priority.^23^

In general, if an automotive company has to develop an ultra-long-range BEV to meet the needs of some consumers, there are some suggestions. It can focus on developing high energy density batteries and adopt appropriate energy-saving technologies for the vehicle (lightweight body, low wind resistance design, low rolling resistance tires, etc.) to reduce the energy consumption and battery capacity.

### Policy recommendations and conclusion

Range anxiety of BEVs is essentially energy replenishment anxiety, and if the energy replenishment problem is not solved, range anxiety will always exist. Although ultra-long-range BEVs (BEV1000) can alleviate range anxiety to a certain extent, they cannot fundamentally solve the problem. In addition, the ultra-long-range BEV is loaded with more batteries, which significantly increases its DMC and makes its performance in terms of dynamic, maneuverability, braking, safety, and economy performances greatly discounted. This paper argues that there is no need for automotive companies to develop ultra-long range (BEV1000) to solve consumers’ range anxiety.

This paper suggests three possible approaches for addressing the range anxiety of BEV consumers. Firstly, it can develop innovative battery technologies to solve the problem of range decay of BEVs in winter.^24^ For example, innovative battery materials resistant to low temperatures can be developed. The technological development and industrialization of solid-state batteries and metal-air batteries can be accelerated. The problem of battery performance decay can be solved in terms of battery thermal management system efficiency optimization, comprehensive power system energy utilization for winter conditions, battery insertion gun insulation, and pulse heating in charging scenarios.^25^ Secondly, it can develop the technology to accurately estimate the remaining range of BEVs.^26^ For example, it is developing an advanced battery residual accurate estimation method to consider the energy consumption of air conditioning, automatic driving calculation energy consumption, intelligent cockpit energy consumption, and other energy consumption outside the powertrain system to estimate the remaining range accurately. It can give consumers the real remaining range data to remove consumers’ uncertainty about the remaining range. Thirdly, it can accelerate the development of fast-charging technologies and the layout of charging and swapping infrastructure as soon as possible. For example, it can accelerate the promotion of charging infrastructure on a large scale and the popularization of ubiquitous low-power charging piles^27^; it can accelerate the R&D of high-power fast-charging technology and industrial promotion and realize the development of a high-voltage platform for BEVs to shorten the charging time^8^; it can accelerate the development and popularization of battery swapping technology and infrastructure for BEVs.^28^ The rapid development of fast-charging and battery swapping technologies will solve the shortage of range and eliminate the range anxiety of BEVs’ consumers. There is likely no need for consumers to buy ultra-long-range BEVs that have nothing but a long range and high price.

However, there are still certain technical difficulties and industrialization challenges in fast-charging and battery swapping technologies, such as the problem of performance degradation of batteries under fast charging. Moreover, building the replenishment network such as charging and swapping takes some time. Therefore, for some consumers with ultra-long-distance driving needs but prefer electric travel, a specific BEV with an ultra-long range can be developed to meet their needs. It should be noted that the following points as developing suggestions can be considered in the development process. Firstly, a battery with high safety and high energy density can be installed to achieve an increase in AER with relatively less battery capacity. For example, it can develop innovative battery anode and cathode materials and accelerate the R&D of innovative technologies such as solid-state and metal-air batteries.^24^ Secondly, it can fully use the vehicle’s energy-saving technologies, such as reducing the vehicle’s wind and rolling resistance coefficients, designing a lightweight body, and using low-energy air conditioning.^23^ Thirdly, it could use efficient range-extender technologies to supplement energy during the transitional period for the full electrification of vehicles.^29^ For example, developing special energy-efficient range extenders, such as roof solar charging panels, rotor range extender engines, fuel cell engines, high-efficiency dictated hybrid engines, etc., to achieve an extended AER for BEVs with lower energy consumption. However, it should be noted that this method is only a transitional means in the period when innovative batteries have not made breakthroughs and charging and swapping infrastructure has not been popularized.

### Limitation of the study

This paper focuses on vehicle performances, TCO, and consumers’ range anxiety for BEVs with different ranges, explores the necessity for automotive companies to develop BEV with an ultra-long range, and gives policy recommendations to address the consumers’ range anxiety. This study still has a series of limitations. Firstly, this paper’s cumulative probability distribution of daily travel range for consumers was based on the travel characteristics of all vehicle consumers in China. It is not drawn for the travel characteristics of BEVs’ consumers. It has some errors in analyzing the travel characteristics of BEV’s consumers. Secondly, this paper did not analyze their carbon emissions considering that the life cycle carbon emissions have similar trend characteristics to the TCO. It makes the analysis of BEVs with different ranges not comprehensive enough. Thirdly, although this paper suggests solving the range anxiety of BEVs’ consumers, it does not propose corresponding optimization solutions for the planning layout of charging infrastructure. In the future, we will continue to study specific implementation solutions to solve consumers’ range anxiety.

## STAR★Methods

### Key resources table

**Table:** 

REAGENT or RESOURCE	SOURCE	IDENTIFIER
**Deposited data**		

BEV’s parameters and technical specification	This paper	N/A
BEV’s component efficiency, mass and cost parameters	This paper	N/A

**Other**		

Technology evaluation model of the powertrain	Liu et al.^14^ Liu et al.^30^	N/A
Battery degradation model	Geng et al.^31^	N/A

### Resource availability

#### Lead contact

Further information and requests for resources should be directed to and will be fulfilled by the lead contact, Zongwei Liu (liuzongwei@tsinghua.edu.cn).

#### Materials availability

This study did not generate new unique reagents.

### Method details

The integrated model is consisted of three sub-model: (1) Technology evaluation model of the powertrain; (2) Total cost of ownership model; (3) Battery degradation model. The functions, specific composition and calculation logic of each sub-model are described as follows.

#### Technology evaluation model of the powertrain

The vehicle performance (battery capacity, motor peak power, curb mass etc.) and direct manufacturing cost of BEV are simulated by using the Technology evaluation model of the powertrain (TEMP) developed by the Tsinghua Automotive Strategy Research Institute at Tsinghua University.^14^^,^^30^ TEMP is a technology-rich, bottom-up approach model which adopts the physics-based method to simulate the actual working state of the BEVs and calculates the energy consumption under the corresponding test cycle. At the same time, according to the mass sub-model and the component sizing sub-model, each BEV component’s corresponding size and mass can be calculated. Combined with the cost sub-model, the calculation of the direct manufacturing cost (DMC) of BEVs with different electric ranges is realized. Detail model detail, data information and corresponding sources are provided in the supplemental information (Figures S1–S4, Tables S1–S5, and Equations S1–S9).

The simulation process is as follows: Firstly, it should determine the basic parameters of the vehicle, such as vehicle segment, base mass (glider mass), wheelbase, aerodynamic coefficient, rolling resistance coefficient, and inertia coefficient. Secondly, it should set up the corresponding vehicle technical specifications (VTS) such as maximum speed, 0-100 km/h time, and the AER. Thirdly, the vehicle simulation module can calculate the energy consumption and BEV’s technical parameter results. The vehicle technical parameter results include the curb mass and component parameters such as motor parameters (power, mass). Fourthly, the calculated vehicle parameters are substituted into the direct manufacturing cost (DMC) and TCO models to obtain the corresponding results.

#### Total cost of ownership model

TCO model considers vehicle and use costs, which consider the battery degradation characteristics and alternative transport scenarios of BEVs with different electric ranges as shown in Figure S5.^32^^,^^33^ The vehicle cost is related to the purchase cost (MSRP), purchase subsidies, resale value, and battery replacement cost. The use cost relates to the purchase tax, vehicle and vessel tax, insurance, maintenance fee, electricity, and alternative travel cost.

#### Vehicle cost model

Vehicle cost is a one-time cost that includes the MSRP, purchase subsidies in 2022, the resale value, and battery replacement cost, as Equation 1.

(Equation 1) $$C_{V,i}={MSRP}_{i}-S_{N,i}-R_{V,i}+R_{B,i}$$

where i refers to the BEVs with different AERs in this paper.

##### Purchase cost, MSRP

MSRP for BEVs can be obtained by multiplying the BEV’s DMC calculated from the TEMP by 1.5.

##### Purchase subsidies $S_{N}$

$S_{N}$ in 2022 ranges from $1857 to $2571 based on the range according to the subsidy standard.^34^ The BEV could obtain $1857 when the AER ranges from 300 km to 400 km. The BEV could obtain $2571 when the AER is above 400 km.

##### Resale value RV

The resale value can be described as follows:

(Equation 2) $$RV=\left\{ \begin{array}{c} \frac{{\left(1-VDR\right)}^{Y}*MSRP}{{\left(1+r\right)}^{Y-1}}\left(Y<X\right)\\ \frac{{\left(1-VDR\right)}^{Y}*{MSRP}_{non-battery}}{{\left(1+r\right)}^{Y-1}}+\frac{{\left(1-VDR\right)}^{Y-X+1}*{MSRP}_{battery}}{{\left(1+r\right)}^{Y-X}}\left(Y\geq X\right) \end{array}\right.$$

*Y* is the lifetime of vehicle ownership, and this paper considers different lifetimes from 1 year to 15 years in TCO analysis. *X* is the year when replacing the battery. It can be calculated for BEVs with different AERs, which will be described next. *r* refers to the discount rate, and it is set at 4%.^35^ *VDR* represents the current vehicle’s annual depreciation rate and is assumed to be 25% for B-segment BEVs. The value is referred the resale value of BEVs in the first year, such as BYD HAN (20.64%), GAC AION V (24.86%), NIO ES6 (25.02%), GAC AION S (27.59%) in 2021 from the China auto dealers chamber of commerce (CADCC).^36^ BEV’s resale value rate in different years can be seen in Figure S5B. As we know, the battery capacity becomes degraded with increasing use time as shown in Figure S5C. The BEV will replace the battery in its lifetime if the degradation of battery performance is too heavy to meet the vehicle performance requirement. In this paper, it is assumed that the resale value rate of the battery is equal to that of the vehicle. RV for the BEVs has two conditions such as without battery replacement (Y<X) and with battery replacement (Y≥X). There would exist the situation that the BEV with a lower AER would have a higher resale value than BEV with a higher AER. It is because the battery replacement makes the BEV with lower AER own the higher battery resale value.

##### Battery replacement cost $R_{B}$

The battery degradation states for BEVs with different electric ranges can be calculated by the battery degradation model. It can be seen that BEV300, BEV400, BEV500, and BEV600 need replace its battery in the 8th, 10th, 12th, and 13th years (considering the round number year), respectively. The battery replacement cost is equal to the battery purchase cost subtracting the battery resale cost, as shown in Equation 3:

(Equation 3) $$R_{B}={MSRP}_{battery}-\frac{{\left(1-VDR\right)}^{Y}*{MSRP}_{battery}}{{\left(1+r\right)}^{Y-1}}$$

where ${MSRP}_{battery}$ is the initial cost of the replaced battery. It assumes that the initial cost of the replaced battery is equal to the original battery cost due to the complexity of predicting battery cost.

#### Use cost model

Use cost is the sum of vehicle purchase tax, vehicle and vessel tax, registration fee, insurance, maintenance fee, electricity cost, and alternative transport cost as follows:

(Equation 4) $$C_{U,i}=T_{P,i}+T_{V,i}+F_{R,i}+I_{i}+C_{M,i}+C_{E,i}+C_{A,i}$$

where $C_{U,i}$ represents *i* kind of BEV with a specific AER.

##### Vehicle purchase tax $T_{P}$, vehicle and vessel tax $T_{V}$

The Chinese government does not impose purchase tax or vehicle and vessel tax on the BEVs to promote their sales to date.^37^ Thus, the Vehicle purchase tax $T_{P}$, vehicle and vessel tax $T_{V}$ are 0 in the calculation process.

##### Registration fee $F_{R}$

The registration fee contains a vehicle checking fee (28.6 $/year) and a license plate fee ($28.6). This paper assumes that BEV’s license plate could be obtained without limitations.

##### Insurance I

Insurance includes compulsory accident liability insurance (135.7 $/year), third-party liability insurance (132$/year), vehicle loss insurance (its value is equal to MSRP∗ 0.88%+$48.9), and deductible-exempt insurance (its value is equal to 20%∗ third-party liability insurance+ 20% ∗ vehicle loss insurance). After calculation, the insurance for BEV300 is 680 $/year.

##### Maintenance fee $C_{M}$

The maintenance fee is calculated based on the distance traveled by the vehicle. The average maintenance fee is 0.01144$/km for BEVs, according to driver survey data from the U.S. and China.^32^

##### Electricity cost $C_{E}$

This paper considers the consumer’s charging habits (charging methods such as private charging pile, public charging pile, and charging pile at the working place) to calculate the electricity cost for BEVs as follows:

(Equation 5) $$C_{E}=\sum_{y=1}^{Y}\sum_{j=0}^{2}\left[\frac{\rho_{j}*{P_{e,j}*EC}_{BEV}*{AVKT}_{y}}{{\left(1+r\right)}^{y}*100}\right]$$

where $\rho_{j}$ represents the probability of using a particular charging method j, the value for private charging pile, public charging pile, and charging pile at the working place is 59%, 24%, and 16%, respectively^33^; $P_{e,j}$ represents the cost of that charging method, the corresponding value obtained by averaging the charging cost at different cities (such as Beijing, Guangzhou and Shanghai) is $0.081, $0.281, and $0.119, respectively. ${EC}_{BEV}$ refers to the electricity consumption for BEV with the specific AER calculated by TEMP, kWh/100 km. ${AVKT}_{y}$ is the annual vehicle kilometer travelled at *y* year, km. According to the *Annual report on analysis of Actual Road travel and fuel consumption of Passenger vehicles in China* formulated by the ICET, the AVKT for B-segment BEV is 15646 km.^38^

##### Alternative transportation cost $C_{A,i}$

If BEVs don’t have sufficient range to meet long-distance travel demands, consumers must use alternative transportation for those trips. The travel probabilities of daily vehicle kilometers traveled can be seen in Figure S5D. The travel probability is about 1.38% when the daily vehicle kilometers traveled is over 300 km. The travel probability is 0 when the daily vehicle kilometers traveled is over 400 km. Thus, the owner of BEV300 still has range anxiety and must use alternative transportation for those trips according to the daily vehicle kilometer traveled probability. First, we should identify the extra range when the travel range is more than 300 km. In this paper, we propose a concept of average probability range to describe the average travel range when the travel range is more than 300 km at different travel probabilities, as shown in Equation 6:

(Equation 6) $$S_{ex}=\frac{\int_{S_{range},0}^{1000}f\left(S_{range}\right)dS_{range}}{f\left(S_{range}\right)-f\left(1000\right)}$$

where $S_{ex}$ represents the average extra travel range, km. $f\left(S_{range}\right)$ is the travel probability function of the travel range as shown in Figure S5D. This paper assumes that 1000 km is the maximum range for BEV in one day because the travel probability is 0. According to the travel probability function, the result of $S_{ex}$ for BEV300 is 54 km. It means there is a 1.38% probability of traveling 354 km in one day in one year. Thus, we can calculate that 5 days might travel more than 300 km for BEV300 in one year.

Second, we assume that when the actual AER of BEV cannot meet the consumer’s daily travel demand, consumers will use alternative transportation for all their daily trips. Considering that a rental car (Internal combustion engine vehicle, ICEV) is more convenient than taking a taxi or public transport for consumers, this paper only adopts the rental car as the alternative transportation method. The alternative transportation cost is shown in Equation 7:

(Equation 7) $$C_{A,y}=Day*P_{rent,y}+\frac{Day*354}{100}*FC*P_{gas,y}$$

where Day is 5 for BEV300 in one year. $P_{rent,y}$ refers to the rental fee for *y* year, it is 57.14$/day in China and we assume it is a constant in different years. FC is the fuel consumption for rental ICEV, we assume it is 7.03 L/100 km.^39^ $P_{gas,y}$ refers to the gas price we should pay to the rental car company at *y* year, we assume that it is a constant (1.14$/L) at different years which is up to the rental car company. Although it constantly changes in recent years in China.

#### Battery degradation model

In this paper, the kind of battery used in the BEVs is NCM Li-ion battery due to its high energy density. Li-ion battery capacity fade includes two parts: cycle aging and calendar aging. Cycling aging is the capacity fade resulting from the charge-discharge process of the battery in use. It is proportional to the full equivalent cycle or Ah throughput (the amount of charge transferred by the battery in cycling). The main factors influencing cycle aging include C-rate and DOD.^40^^,^^41^ Our group has established the cycle aging model based on the battery degradation model developed by NREL.^31^ The calculation of capacity loss caused by cycle aging is shown in Equations 8, 9, and 10:

(Equation 8) $$d_{eff}={\left(\frac{D_{A}}{D_{R}}\right)}^{u_{0}}*\exp\left(u_{1}*\left(\frac{D_{A}}{D_{R}}-1\right)\right)*\left(\frac{C_{R}}{C_{A}}\right)*d_{actual}$$

(Equation 9) $${Q_{loss}}_{percycle}\left(n\right)=\frac{d_{eff}}{L_{R}*D_{R}*{Ca}_{R}}*EOL_{rate}$$

(Equation 10) $$Q_{loss_{cyc}}\left(t\right)=Q_{loss_{percycle}}*f_{cycle}*t$$

where $d_{eff}$ is the effective Ah-throughput per cycle of batteries; $D_{A}$ is the actual DOD of batteries. We assumed that all the BEVs have the same DOD and their values are 90%; $D_{R}$ is the rated DOD of batteries under rated operating conditions, corresponding to the rated battery cycle life, which is set as 80%.^42^ $C_{A}$ is the actual C-rate of batteries; $C_{R}$ is the rated C-rate of batteries under rated operating condition, corresponding to the rated battery cycle life, which is set as 1 C in this paper $d_{actual}$ is the actual Ah-throughput per cycle of batteries^41^; ${Q_{loss}}_{percycle}$ is the capacity loss caused by cycle aging per cycle of batteries; $L_{R}$ is the rated cycle life of batteries under rated operating condition; ${Ca}_{R}$ is the battery capacity, Wh/kg. $EOL_{rate}$ is the rated SOH of batteries representing the end of battery life, which is set as 80% in this paper; $f_{cycle}$ is the annual use frequency of batteries, it can be calculated through dividing the annual travel miles (15686 km/year in this year) by the AER of BEV; $Q_{loss_{cyc}}\left(t\right)$ is the capacity loss caused by cycle aging per year of batteries.

Calendar aging is mainly up to irreversible self-discharge capacity loss caused by the lithium loss during solid-state-interphase formation on the negative electrode. The operating conditions of the battery in the vehicle are relatively stable due to the cooling system. The influence of temperature on calendar aging can be ignored in this paper. Our group has established the calendar aging model, which is demonstrated to be proportional to the square root of time as shown in Equation 11:

(Equation 11) $$Q_{loss_{cal}}\left(t\right)=C*\sqrt{t}$$

where $Q_{loss_{cal}}\left(t\right)$ is the calendar aging at *t* year. Parameter C is a given battery’s calendar aging characteristic parameter, referring to the capacity loss after the first five years of placement.

This paper assumes that BEVs charge only when the battery energy runs out. At the same travel miles (such as 15646 km/year), BEVs with different AERs would have different charge-discharge cycles. The longer the BEV’s AER is, the lower the charge-discharge cycle is. For the BEV with the specific AER, its battery capacity fade is calculated as the sum of capacity loss caused by cycle and calendar aging, as shown in Equation 12.

(Equation 12) $${Q_{loss}\left(t\right)=Q}_{loss_{cyc}}+Q_{loss_{cal}}$$

## Data Availability

All underlying data used in this paper is available in the main text or the supplementary information or its sources have been clearly stated. Any additional information required to reanalyze the data reported in this paper are available from the lead contact upon request.
