# Supplementary material for: Comprehensive assessment for different ranges of battery electric vehicles: Is it necessary to develop an ultra-long range battery electric vehicle?
Source: iScience. 2023 Apr 20;26(6):106654. doi: 10.1016/j.isci.2023.106654 (PMC10199263; doi:10.1016/j.isci.2023.106654)
Supplement: Document S1. Figures S1–S5 and Tables S1–S5 [file mmc1.pdf]

iScience, Volume *26*

Supplemental information

Comprehensive assessment for different ranges of battery electric vehicles: Is it necessary to develop an ultra-long range battery electric vehicle?

Xinglong Liu, Fuquan Zhao, Jingxuan Geng, Han Hao, and Zongwei Liu

The descriptions of TEMP and TCO models

The TEMP and TCO models are shown in **Figure S1**. TEMP is divided into four sub-models: energy consumption sub-model, mass sub-model, powertrain component sizing sub-model, and DMC sub-model. The steps for powertrain technologies evaluation using TEMP are as follows.

Firstly, it should determine the basic parameters of the vehicle, such as vehicle segment, base mass (glider mass), wheelbase, aerodynamic coefficient, rolling resistance coefficient, and inertia coefficient. Secondly, it should set up the corresponding vehicle technical specifications (VTS) such as maximum speed, 0-100km/h time, and the AER. Thirdly, the vehicle simulation module can calculate the energy consumption and BEV's technical parameter results. The vehicle technical parameter results include the curb mass and component parameters such as motor parameters (power, mass). Fourthly, the calculated vehicle parameters are substituted into the direct manufacturing cost (DMC) and TCO models to obtain the corresponding results.

There are two main innovations in methodology: first, the TEMP model adopts the physics-based method to simulate the actual working state of the BEVs and calculates the energy consumption under the corresponding test cycle. At the same time, according to the mass sub-model and the component sizing sub-model, each BEV component's corresponding size and mass can be calculated. Combined with the cost sub-model, the calculation of the direct manufacturing cost (DMC) of BEVs with different electric ranges is realized. Second, the TCO model considers vehicle and use costs, which consider the battery degradation characteristics and alternative transport scenarios of BEVs with different all-electric ranges (AER). Innovations in these two aspects can significantly improve the accuracy and authenticity of the TCO of BEVs. Based on methodological innovations, this study can answer whether it is necessary to develop ultra-long-range BEVs.

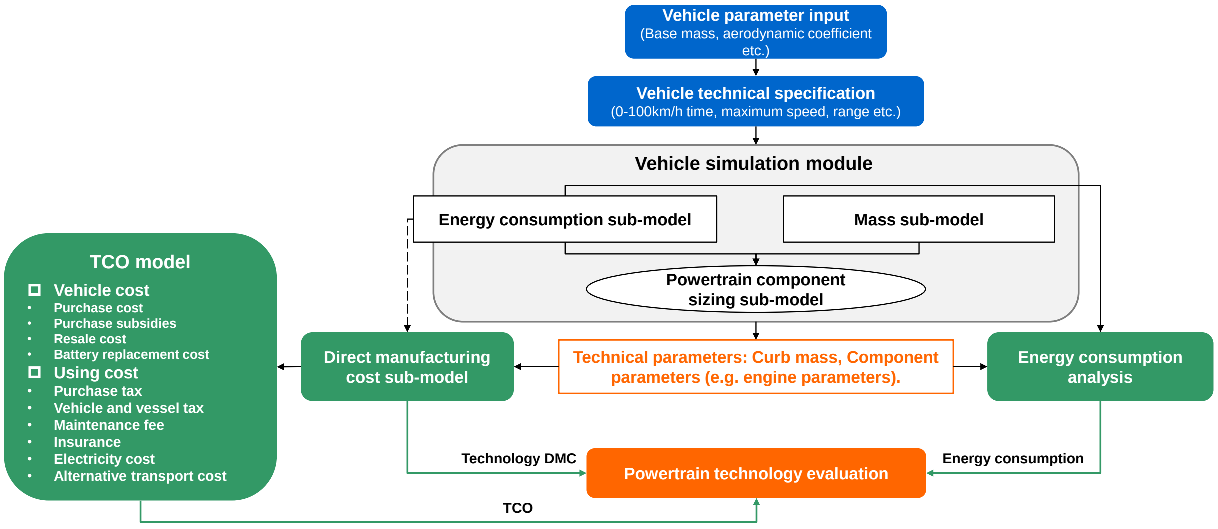

Figure S1. The technology evaluation model of the powertrain (TEMP) and TCO model, Related to STAR Methods.

TEMP model

Basic assumptions

The basic assumptions include vehicle parameters, VTS, and vehicle test driving cycles. It should note that although this research is mainly based on China's BEV market and consumer preferences, the results and discussion are also applicable to other countries since BEVs have similar performance parameters and functional characteristics in the world. The B-segment vehicle with 2700-2900mm wheelbase is selected to be studied. The classification of the vehicles segment is based on the standard formulated by the China Automotive Technology and Research Center (CATRC), which is representative of China's market [1]. The basic vehicle parameters are obtained through the statistics and analysis of the B-segment vehicles in the Chinese market, as shown in **Table S1**.

Table S1 Basic vehicle parameters, Related to STAR Methods.

Vehicle type	B-segment vehicle
Glider mass (kg)	1350
Wheelbase (mm)	2795
Aerodynamic coefficient (-)	0.30
Rolling resistance coefficient (-)	0.01
Inertia coefficient (-)	1.05
Vehicle frontal cross-sectional area (m^2)	2.25

As abovementioned, the sizes of BEV's components are related to the VTS. To size individual components, the first step is to define the VTS. **Table S2** shows the VTS of BEVs according to the vehicle performance of current vehicles on sale in the Chinese market and the technical specifications defined by national standards [2,3]. The basic

dynamic performance is that the acceleration time is from 0 to 100km/h in 9s. Basic dynamic performance is the only performance index to determine the drive motor power of the BEVs.

Table S2 BEV's performance categories, Related to STAR Methods.

Vehicle technical speciation		Value
Basic dynamic performance	0-100km/h time (s)	9
BEV	AER (km)	>200

Calculating method of different sub-models

It is well known that test-driving cycles significantly impact vehicle energy consumption for vehicles [4]. The China light-duty vehicle test cycle (CLTC) used in China is shown in **Figure S2**. CLTC is formulated by China Automotive Technology & Research Center Co. Ltd according to the actual driving characteristics of vehicles used in China [5]. According to the energy consumption test standards for BEVs in China, the CLTC is required to adopt to test the energy consumption of BEVs [6].

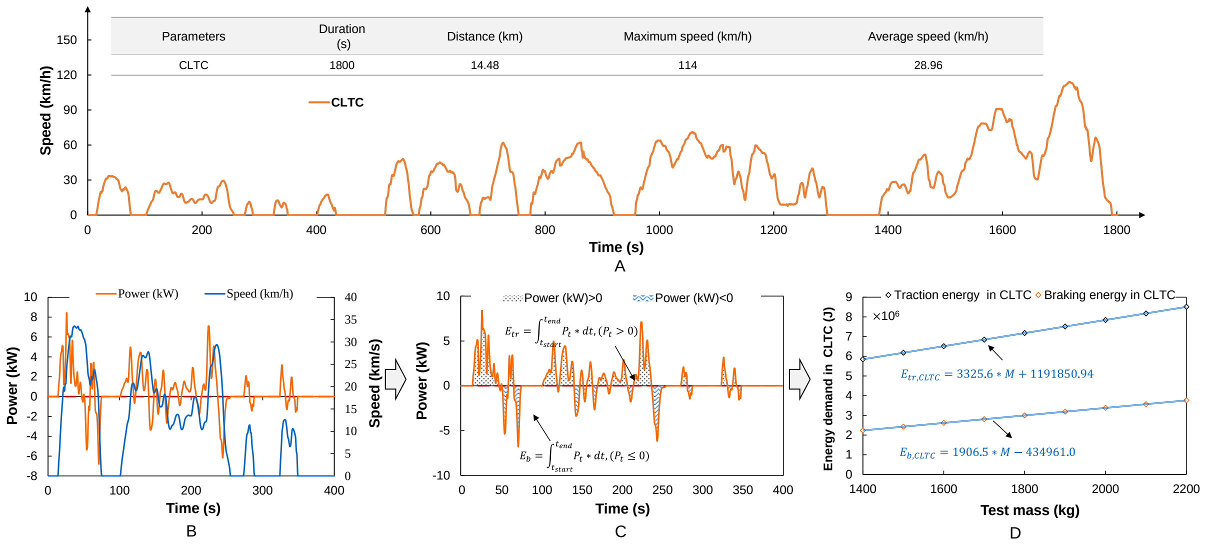

Figure S2. The CLTC test driving cycle and the method to calculate the vehicle energy demand of CLTC driving cycles : (A) CLTC test driving cycles;(B) power and speed of the CLTC cycle during the first 400 seconds; (C) traction energy (E_{tr}) and break energy (E_b) during the first 400 seconds of the CLTC; (D) the energy demand related to the test mass of the test driving cycles, Related to STAR Methods.

Energy consumption sub-model

The paper builds a physics-based energy consumption calculation sub-model to simulate the BEVs' energy flow and calculate the energy consumption under the CLTC driving cycle. First, calculate the energy demand for completing a CLTC driving cycle; Second, calculate the BEVs' the electricity consumption based on the work principles and vehicle component efficiencies [7,8].

Firstly, the dynamic vehicle equation obtains the longitude force for driving a vehicle, as shown in **Equation S1**. Secondly, the vehicle power requirement could be multiplied by force obtained from **Equation S1** to the vehicle speed. Finally, the energy demand under a specific CLTC cycle could be obtained by integrating the power requirement by time, as **Equation S2** shows. BEVs can recover some e energy during deceleration due to the braking energy recovery functions [9]. Therefore, when calculating the energy demand of BEVs, the regenerative energy needs to be removed [10].

$$F_t = M * g * fcos\alpha + M * g * sin\alpha + \frac{Cd * A * {v_t}^2}{21.15} + \delta M * a_t \rightarrow P_t = F_t * v_t \qquad \text{(Equation S1)}$$

Where F_t denotes the longitude force to drive the vehicle at *t* moment, N； *M* is the test mass, kg. It refers to the sum of the mass in running order, the mass of optional fitted equipment to that specific vehicle, and a certain proportion of the maximum vehicle load [8,11]. The proportion of the vehicle load is equal to 0.15 in the case of passenger cars [11]. g is the gravitational acceleration, m/s^2. *f* is the rolling resistance coefficient. *Cd* is the aerodynamic coefficient. *A* is the vehicle's frontal cross-sectional area, m^2. α is grade. It is assumed 0 when the BEVs are tested under the CLTC. δ is a number greater than one to account for rotating inertia. v_t is the vehicle speed at time *t*, m/s. a_t is the accelerated speed at time *t*, m/s^2. P_t is the power required to drive the vehicle at time *t*, W.

$$E = \int_{t_{start}}^{t_{end}} P_t * dt = \sum_{t_{start}}^{t_{end}} E_t, \begin{cases} E_t = E_{tr} = P_t * t,\ (P_t > 0) \\ E_t = \mu E_b = -P_t * t * \mu\ (P_t \leq 0) \\ \mu = R_{rb} * n_{rb} * n_{e-dl} * n_m * n_{mc} * n_b \end{cases} \qquad \text{(Equation S2)}$$

E is the energy demand when the BEV is driving under the CLTC, J. E_{tr} is the traction energy, J. E_b is the energy during braking, J. μ is the regenerative braking efficiency. Its value is determined by the upper limit of the braking energy recovery (R_{rb}), the regenerative braking ratio (n_{rb}), the driveline system efficiency (n_{e-dl}), the motor efficiency (n_m), the motor controller efficiency (n_{mc}) and the battery charging efficiency (n_b). The braking energy recovery exists within the upper limit (R_{rb}) because it cannot realize the full recovery of breaking energy. The reasons are that the highest critical state of charge (SOC) of the battery and the minimum vehicle speed of vehicle deceleration would reduce the proportion of braking energy recovery [12-14]. Regenerative braking ratio (n_{rb}) refers to the ratio of regenerative braking to the total braking energy when the vehicle brakes [15].

Figure S2C depicts the speed curve and required power with a specific mass during the first 400 s of the CLTC. The calculation equations of traction energy and braking energy can be seen in the figure. The traction and braking energy are obtained by integrating the traction and braking power by time. **Figure S2D** shows the vehicle energy demand with different test masses under the CLTC driving cycle. The energy demand is linearly related to the test mass when the other basic parameters (rolling resistance coefficient, aerodynamic coefficient, vehicle frontal cross-sectional area,

etc.) are consistent. The resulting equations of energy demand versus test mass under CLTC driving cycles are the input to the powertrain component sizing sub-model.

The electricity consumption for BEVs can be calculated using **Equation S3**.

$$EC = \frac{100 * E}{n_{ce} * 3.6 * 10^6 * S} \quad \text{(Equation S3)}$$

Where EC indicates the electricity consumption, kWh/100km.

Table S3 summarizes the critical efficiency parameters of BEVs in 2022. The data are collected from the real parameter of the vehicles on sale in the Chinese market, the Technology Roadmap for Energy-saving and New Energy Vehicles 2.0 formulated by the China Society of Automotive Engineering (SAE-China), and another research literature [16-19].

Table S3 BEV's component efficiency parameters and data, Related to STAR Methods.

Description	BEV
Battery charge and discharge efficiency n_b [16]	95%
Reducer efficiency n_r [19]	95%
Electric motor efficiency n_m [19]	95%
Motor controller efficiency n_{mc} [19]	95%
Upper limit of the braking energy recovery R_{rb} [20]	80%
Regenerative braking ratio n_{rb} [13]	75%

Mass sub-model

The vehicle mass sub-model of BEV's is shown in **Equation S4**:

$$m_{curb\ mass} = \sum_{co=1}^{h} m_{co} \quad \text{(Equation S4)}$$

Where $m_{curb\ mass}$ denotes the curb mass of the BEV, kg. m_{co} indicates the component mass. h indicates the total number of components. BEVs have five components: battery system, motor, motor controller, reducer, and glider. In this paper, m_m is the mass of the electric motor system. To calculate its mass, we introduce two power densities, including motor power density (k_{m1}), motor controller power density (k_{m2}). The motor mass of BEVs can be determined by motor power density and motor controller power density. The electric motor system of BEVs generally forms an electric drive system with a reducer. The mass of the electric drive system of BEVs is determined by the k_{m3}. m_b is the mass of the battery system, and it is the function of energy density (k_b). $m_{charger}$ is the mass of on-board charger and its value is a constant. The main mass parameters of components in 2022 are shown in **Table S4.**

Table S4 BEV's component mass parameters and data, Related to STAR Methods.

Component	Characteristic parameters	Data
Electric motor system m_m [16]	k_{m1} (kg/kW)	4

	k_{m2} (kg/kW)	20.33
	k_{m3} (kg/kW)	1.85
Battery system m_b [16]	k_b (kg/kWh)	135
Charger $m_{charger}$ [18]	Constant (kg)	5
Glider m_{glider}*	Constant (kg)	1350

Component sizing sub-model

Based on the energy consumption sub-model and mass sub-model, it can calculate the component sizes of BEVs according to the VTS. Sizing each component for each vehicle is an iterative process [21]. **Figure S3** describe the iterative process For example, increasing the AER of BEVs would increase the required battery energy, which increases vehicle mass and energy consumption. To achieve the same performance specification (e.g., 0-100km/h accelerating time), BEVs need to increase the peak powers of motor. In turn, increasing the motor peak power will increase the vehicle curb mass and then affect the required battery energy to achieve the target AER. Therefore, the calculation of component size needs an iterative solution until all the performance specifications are met.

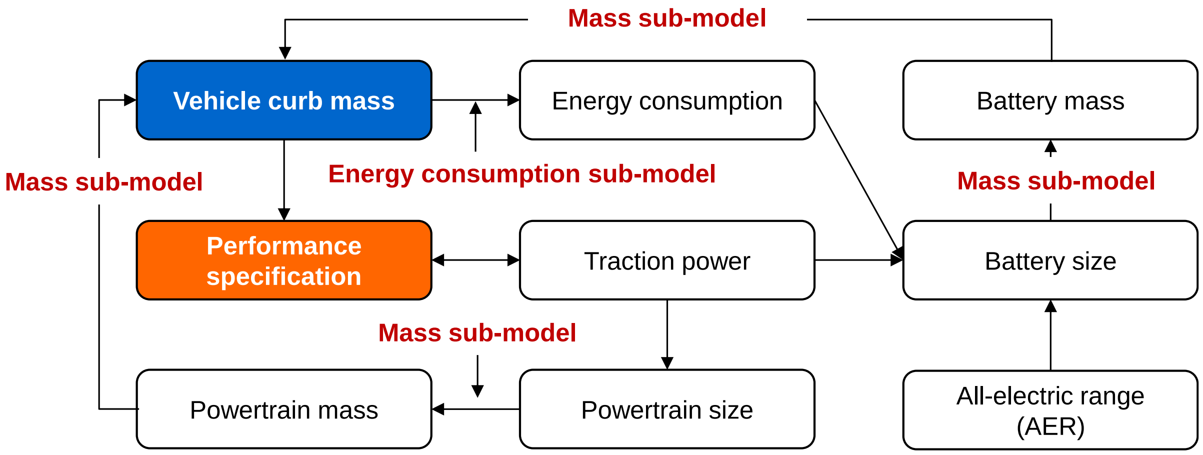

Figure S3. The schematic diagram of the component sizing model for BEVs, Related to STAR Methods.

This paper constructs a set of solving equations to calculate the component size of BEVs, as shown in **Equation S5**.

$$\begin{cases} \frac{1}{\alpha} * P_m = \frac{V_a}{3600 * n_r}(\delta * M * 0.5 * V_a/(3.6 * t_a) + M * g * f + \frac{Cd * A * v_a^{\ 2}}{21.15}) \\ Q = \frac{S_{target} * (E_{tr} - \mu * E_b)}{36000000 * S * n_b * n_m * n_{mc} * n_{m-dl} * (SOC_{max} - SOC_{min})} \\ M = m_{glider} + P_m/k_{m3} + m_{charger} + Q/k_b + 100 + 75 \end{cases} \quad \text{(Equation S5)}$$

Where P_m is the peak electric motor power, kW. V_a is the speed of 100km/h. n_r is the reducer efficiency. t_a is the accelerating time from 0 to 100km/h, s. α is the excess coefficient. It refers to that part of the peak power of the BEV's powertrain is

used to ensure vehicle performance. In contrast, the other part is provided for the energy supply for accessories on the vehicle, such as the air conditioner. According to the design principle of vehicle powertrains, the value of α is generally 1.1-1.2 [22]. In this paper, the value of α is assumed to be 1.2. Where Q is the required battery energy for BEVs to reach the target AER, kWh. S_{target} is the target AER of BEVs, km. SOC_{max} is the maximum state of charge of the BEVs battery when in service, it is 1 in this paper. SOC_{min} is the minimum state of charge of the BEVs battery when in service, it is 0.1 in this paper. The mass in the running order is the sum of curb mass and 100kg. The value of the mass of optional fitted equipment to that specific vehicle, and a certain proportion of the maximum vehicle load are generally 75kg for light-duty passenger vehicles.

DMC cost sub-model

The paper builds a bottom-up model to calculate the BEV's DMC. The calculation equation is shown in **Equation S6**.

$$c_{BEV} = \sum_{co=1}^{h} c_{co} \qquad \text{(Equation S6)}$$

Where c_{BEV} indicates the BEV's DMC, \$. c_{co} is the DMC of component co, \$. The DMC of BEVs includes electric motor cost (c_{motor}), regenerative braking system cost (c_{break}), battery cost (c_b), motor controller cost ($c_{control}$), reducer cost ($c_{reducer}$), on-board charger cost ($c_{charger}$) and glider cost (c_{glider}).

The basic cost data are collected from different countries at different years. To keep comparability, all the cost data are standardized based on the U.S. dollar value in China 2020 through the average inflation rate, average exchange rate, and price level ratio of purchasing power parity conversation factor to the market exchange rate to keep comparability. **Equation S7** is usually used to standardize values in different countries and different years [23].

$$C_{2020,CN} = [C_{y,A} \times \prod_{y}^{2020} IR_y \times (L_{y,CN}/L_{y,A})]/ER_{2020,CN/U.S.} \qquad \text{(Equation S7)}$$

Where $C_{t,CN}$ denotes the standardized value in China 2020. $C_{y0,A}$ denotes the value in country *A* currency and year y. IR_y denotes the inflation rate of country *A* and year y. $L_{y,CN}$ and $L_{y,A}$ denotes the price level ratio of purchasing power parity conversion factor to the market exchange rate of China and country *A* in 2020, respectively. $ER_{2020,CN/A}$ denotes the China currency against the U.S. dollar exchange rate in 2020. Macroeconomic parameters for calculation could be found in International Monetary Fund and World Bank [1,6,23].

Brake energy recovery is an essential energy-saving technology for BEVs. The DMC of regenerative braking system plays a vital role in the total cost of electrified vehicles. Limpan et al. found that the design of a regenerative braking system is related to the curb mass of vehicles [24]. This paper considers the curb mass as the characteristic parameter to calculate the DMC of the regenerative braking system. According to the

disassembly data of EPA [25], the cost model of the regenerative braking system is fitted as shown in **Equation S8**:

$$c_{break} = 0.017 * m + 142.14 \quad \text{(Equation S8)}$$

Glider refers to the remaining part of the vehicle after removing the parts related to the powertrain. The development of electrification and vehicle intelligence makes the glider part of various powertrain technologies more and more similar. The glider of different powertrain technologies would converge with the BEV's glider in the future. Based on the statistics of BEVs in the current Chinese market, the manufacturer's suggestion retail price of the glider is determined [26]. According to the retail price equivalent coefficient method, the manufacturer suggested retail price is generally 1.5-2 times the DMC of the vehicle [27]. This paper assumes selecting 1.5 as the retail price equivalent coefficient to calculate the glider DMC. Taking the glider mass as characteristic parameter, the relationship between glider DMC and glider mass is linearly fitted as shown in **Equation S9**.

$$c_{glider} = 0.6 * m_{glider} + 344.6 \quad \text{(Equation S9)}$$

The DMC of the motor system and battery system is obtained by linear scaling of its unit cost according to the characteristic parameters. The DMC of the on-board charger, power distribution, and harness system is generally constant. The values for these components are collected from another research [18,28]. **Table S5** summarizes the cost parameters of BEV in 2020.

Table S5 BEV's component cost parameters and data, Related to STAR Methods.

Component	Description	Data
Motor system c_m and c_{mc} [16]	Motor cost ($/kW)	5.00
	Motor controller cost ($/kW)	5.71
Battery system c_b [16]	Power- and energy-based battery ($/kWh)	214.29
Charger $c_{charger}$ [18]	On-board charger cost ($)	284.00
Power Distribution and harness System c_{pdhs} [28]	Compared with ICEV, the increased power distribution and control system for BEVs ($)	298.43

2.1.3 Model validation of TEMP

This section mainly verifies the TEMP by comparing the output results (the curb mass, DMC, and comprehensive energy consumption as evaluation indexes) with the corresponding parameters of the vehicles in the current market. The data (curb mass, DMC, and comprehensive energy consumption) of vehicles are collected from the well-known automotive information website (automotive home) and recommended models for the promotion and application of the NEVs catalog released by the Ministry of Industry and Information Technology [29,30]. We can only obtain the manufacturer's suggested retail price from the automobile home. This paper assumes to select 1.5 as the retail price equivalent coefficient (the ratio of MSRP to DMC) to calculate the

vehicle DMC [31,32]. **Figure S4A and B** show the curb mass and DMC distribution related to 2700-2900mm wheelbase, respectively.

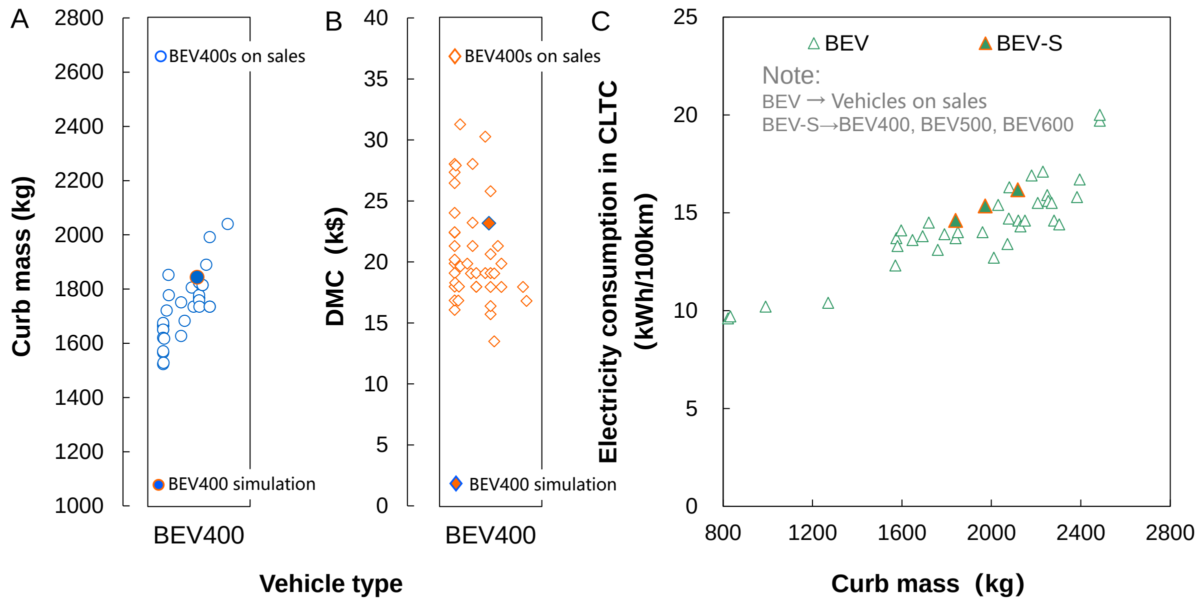

Figure S4. Validation for TEMP: (A) Curb mass; (B) DMC; (C) electricity consumption (EC) under the CLTC driving cycles, Related to STAR Methods.

The BEV curb mass results are relatively higher than the average curb mass of corresponding vehicles on sale. The main reason is that the battery system density (160Wh/kg) we selected is relatively low according to the industry's average [33,34]. It would lead to the battery system density being lower than that of the vehicles on sale. As shown in **Figure S4B**, the DMC results of BEV are higher than the average DMC of vehicles on sale. The reason can be explained that the selected battery unit cost from the Technology Roadmap for Energy-saving and New Energy Vehicles 2.0 would be higher than that of the vehicles on sale due to the battery unit cost declining gradually. This paper also compares the electricity consumption of BEVs calculated by TEMP with that of the vehicles on sale in the market, as shown in **Figure S4C**. The electricity consumption of BEVs in CLTC is within the electric range of the vehicles available in the market. In general, the curb mass, DMC, and electricity consumption are all in the corresponding ranges of the vehicles on sale. It indicates the validity of TEMP to evaluate the BEVs' curb mass, DMC, and electricity consumption.

TCO model and battery degradation model

The corresponding results of TCO model are illustrated in **Figure S5**. The model adopted in this paper examines two aspects contributing to vehicle TCO, such as vehicle cost and use cost [35,36]. The vehicle cost is related to the purchase cost (MSRP), purchase subsidies, resale value, and battery replacement cost. The use cost relates to the purchase tax, vehicle and vessel tax, insurance, maintenance fee, electricity, and alternative travel cost.

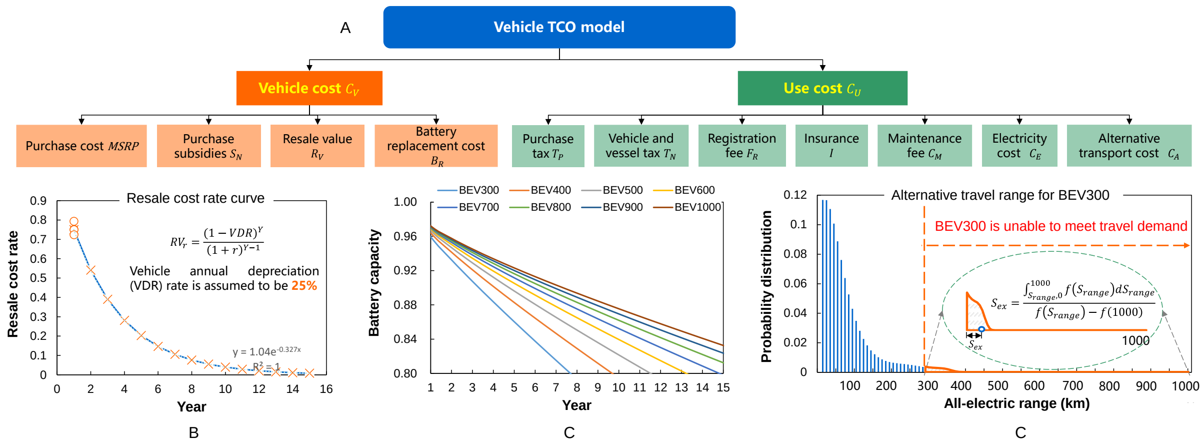

Figure S5. The TCO model framework for BEV: (A) model framework; (B) BEV's resale value rate in different years; (C) battery degradation models for BEVs with different ranges; (D) Alternative travel cost for BEV300, Related to STAR Methods.

BEV's resale value rate in different years can be seen in **Figure S5B**.

The battery capacities of BEVs with different AERs become degraded with increasing use time as shown in **Figure S5C**.

The travel probabilities of daily vehicle kilometers traveled can be seen in **Figure S5D**.

Reference

1. Automotive Data of China co.,Ltd. China automobile low carbon action plan (CALCP) research report 2021 [R]. Beijing, 2021. https://www.chinacace.org/news/uploads/2021/07/1627352257602220.pdf.
2. National Technical Committee on Automobile of Standardization Administration. (2005). GB/T 19752-2005 Hybrid electric vehicle performance test method [S]. Beijing: Standards Press of China.
3. Iora, P., and Tribioli, L. (2019). Effect of Ambient Temperature on Electric Vehicles' Energy Consumption and Range: Model Definition and Sensitivity Analysis Based on Nissan Leaf Data. World Electric Vehicle Journal *10*. 10.3390/wevj10010002.
4. S. Tsiakmakis, Fontaras, G., Cubito, C., Pavlovic, J., Anagnostopoulos, K., and Ciuffo, B. (2017). From NEDC to WLTP: effect on the type-approval CO2 emissions of light-duty vehicles. JRC sicence for policy report. 10.2760/9341910.2760/35344.
5. Zhao, F., Liu, X., Zhang, H., Liu, Z., and Zhang, W. (2022). Automobile Industry under China's Carbon Peaking and Carbon Neutrality Goals: Challenges, Opportunities, and Coping Strategies. Journal of Advanced Transportation *2022*, 1-13. 10.1155/2022/5834707.
6. National Technical Committee on Automobile of Standardization Administration. (2021). GB/T 18386.1-2021 Test methods for energy consumption and range of electric vehicles—Part 1:Light-duty vehicles [S]. Beijing: Standards Press of China.
7. Innovation Center for Energy and Transportation. (2018). Annual report on analysis of Actual Road travel and fuel consumption of Passenger vehicles in China. http://www.icet.org.cn/reports.asp. 10.3969/j.issn.1673-3142.2020.04.029.
8. Ministry of Finance, Tax administration, Ministry of Industry and Information Technology, Ministry of Science. (2021). Announcement on exemption of new energy vehicle purchase tax.
9. Ou, S., Hsieh, I.L., He, X., Lin, Z., Yu, R., Zhou, Y., and Bouchard, J. (2021). China's vehicle electrification impacts on sales, fuel use, and battery material demand through 2050: Optimizing consumer and industry decisions. iScience *24*, 103375. 10.1016/j.isci.2021.103375.
10. He, X., Ou, S., Gan, Y., Lu, Z., Przesmitzki, S.V., Bouchard, J.L., Sui, L., Amer, A.A., Lin, Z., Yu, R., et al. (2020). Greenhouse gas consequences of the China dual credit policy. Nature Communications *11*. 10.1038/s41467-020-19036-w.
11. Pavlovic, J., Ciuffo, B., Fontaras, G., Valverde, V., and Marotta, A. (2018). How much difference in type-approval CO2 emissions from passenger cars in Europe can be expected from changing to the new test procedure (NEDC vs. WLTP)? Transportation Research Part A: Policy and Practice *111*, 136-147. 10.1016/j.tra.2018.02.002.
12. Lv, C., Zhang, J., Li, Y., and Yuan, Y. (2015). Mechanism analysis and evaluation methodology of regenerative braking contribution to energy efficiency improvement of electrified vehicles. Energy Conversion and Management *92*, 469-482. 10.1016/j.enconman.2014.12.092.
13. Hu, X. (2022). Preface for Feature Topic on Advanced Battery Management for Electric Vehicles. Automotive Innovation *5*, 105-106. 10.1007/s42154-022-00182-4.
14. Bai, X., Chen, G., Li, W., Jia, R., Xuan, L., Zhu, A., and Wang, J. (2021). Critical Speeds of Electric Vehicles for Regenerative Braking. Automotive Innovation *4*, 201-214. 10.1007/s42154-021-00143-3.

15. Ahmad, F., Saad Alam, M., Saad Alsaidan, I., and Shariff, S.M. (2020). Battery swapping station for electric vehicles: opportunities and challenges. IET Smart Grid *3*, 280-286. 10.1049/iet-stg.2019.0059.
16. Chen, K., Zhao, F., Hao, H., Liu, Z., and Liu, X. (2021). Hierarchical Optimization Decision-Making Method to Comply with China's Fuel Consumption and New Energy Vehicle Credit Regulations. Sustainability *13*. 10.3390/su13147842.
17. Cai, W., Wu, X., Zhou, M., Liang, Y., and Wang, Y. (2021). Review and Development of Electric Motor Systems and Electric Powertrains for New Energy Vehicles. Automotive Innovation *4*, 3-22. 10.1007/s42154-021-00139-z.
18. Ou, S., Yu, R., Lin, Z., Ren, H., He, X., Przesmitzki, S., and Bouchard, J. (2019). Intensity and daily pattern of passenger vehicle use by region and class in China: estimation and implications for energy use and electrification. Mitigation and Adaptation Strategies for Global Change *25*, 307-327. 10.1007/s11027-019-09887-0.
19. Smallbone, A., Jia, B., Atkins, P., and Roskilly, A.P. (2020). The impact of disruptive powertrain technologies on energy consumption and carbon dioxide emissions from heavy-duty vehicles. Energy Conversion and Management: X *6*. 10.1016/j.ecmx.2020.100030.
20. Sun, X., Ouyang, M., and Hao, H. (2022). Surging lithium price will not impede the electric vehicle boom. Joule *6*, 1738-1742. 10.1016/j.joule.2022.06.028.
21. Lipman, T.E., and Delucchi, M.A. (2006). A retail and lifecycle cost analysis of hybrid electric vehicles. Transportation Research Part D: Transport and Environment *11*, 115-132. 10.1016/j.trd.2005.10.002.
22. Liu, Z., Zhang, W., and Zhao, F. (2022). Impact, Challenges and Prospect of Software-Defined Vehicles. Automotive Innovation *5*, 180-194. 10.1007/s42154-022-00179-z.
23. Qiao, Q., Zhao, F., Liu, Z., and Hao, H. (2019). Electric vehicle recycling in China: Economic and environmental benefits. Resources, Conservation and Recycling *140*, 45-53. 10.1016/j.resconrec.2018.09.003.
24. China auto dealers chamber of commerce. Car Preservation Rate Report. 2022.7. http://www.cadcc.com.cn/article/1079.html.
25. Geng, J., Gao, S., Sun, X., Liu, Z., Zhao, F., and Hao, H. (2022). Potential of electric vehicle batteries second use in energy storage systems: The case of China. Energy *253*. 10.1016/j.energy.2022.124159.
26. Zhao, F., Chen, K., Hao, H., Wang, S., and Liu, Z. (2018). Technology development for electric vehicles under new energy vehicle credit regulation in China: scenarios through 2030. Clean Technologies and Environmental Policy. 10.1007/s10098-018-1635-y.
27. Ministry of Finance of the People's Republic of China, Ministry of Science and Technology of the People's Republic of China, Ministry of Industry and Information Technology, National Development and Reform Commission. (2021). Notice on adjust and improve NEVs promotion and application of financial subsidies [Chinese].
28. Wei, W., Ramakrishnan, S., Needell, Z.A., and Trancik, J.E. (2021). Personal vehicle electrification and charging solutions for high-energy days. Nature Energy *6*, 105-114. 10.1038/s41560-020-00752-y.
29. The parameter of vehicel models on sale in the market from Automotive home. 2020. https://www.autohome.com.cn/beijing/.

30. Ministry of Industry and Information Technology of the People's Republic of China. Recommended models for the promotion and application of new energy vehicles catalog. 2022. https://www.miit.gov.cn/jgsj/zbys/wjfb/art/2021/art_9f9083a59a934859884f2739926ccce2.html.
31. FEV (2013). Updated Indirect Cost Multiplier (ICM) Methodology. International Council on Clean Transportation.
32. Liu X, Zhao F, Hao H. (2022). Research on the Calculation Methods of Automobile Technology Cost Base on Chinese Market[C]//Proceedings of China SAE Congress 2020: Selected Papers. Singapore: Springer Nature Singapore.
33. Liu, Z., Hao, H., Cheng, X., and Zhao, F. (2018). Critical issues of energy efficient and new energy vehicles development in China. Energy Policy *115*, 92-97. 10.1016/j.enpol.2018.01.006.
34. Automotive Data of China co.,Ltd. Market research | 2022 power battery market profile. https://mp.weixin.qq.com/s?__biz=MzI5NjYxNzg0NA==&mid=2247584890&idx=1&sn=8cd9cc0a1f5a8bc0e2f30d3fa7ac0636&chksm=ec425e61db35d77715b688ad25945d739f4ff913f616c2b4ddf966cbb3332a12801c321c2c13&scene=27.
35. Hao, X., Lin, Z., Wang, H., Ou, S., and Ouyang, M. (2020). Range cost-effectiveness of plug-in electric vehicle for heterogeneous consumers: An expanded total ownership cost approach. Applied Energy *275*. 10.1016/j.apenergy.2020.115394.
36. Ouyang, D., Zhou, S., and Ou, X. (2021). The total cost of electric vehicle ownership: A consumer-oriented study of China's post-subsidy era. Energy Policy *149*. 10.1016/j.enpol.2020.112023.
